# Congenital Anterior Urethral Diverticulum Presenting with Fluctuant Penile Mass

**Published:** 2013-07-13

**Authors:** Shasanka Shekhar Panda, Minu Bajpai, Rashmi R Das, Manisha Jana

**Affiliations:** Department of Paediatric Surgery, All India Institute of Medical Sciences, New Delhi, India, 110029; Department of Paediatric Surgery, All India Institute of Medical Sciences, New Delhi, India, 110029; Department of Paediatrics, All India Institute of Medical Sciences, New Delhi, India, 110029; Department of Radiology, All India Institute of Medical Sciences, New Delhi, India, 110029

**Dear Sir,**

Congenital anterior urethral diverticulum (CAUD) is an uncommon condition in children. They usually present with penile swelling followed by recurrent urinary tract infection (UTI) and poor urinary stream.[1] It is a cause of lower urinary tract obstruction in males. We present a case of congenital anterior urethral diverticulum that presented with penile mass and UTI, treated successfully by open surgical method.

A 15-month old male child presented with fluctuant ventral penile mass which was noted at birth. Its proximal extent was the penoscrotal junction and involved whole of the penis. It used to become more prominent during micturition. Presenting features also included dribbling of urine, poor urinary stream and urinary tract infection. On compression of the mass, urine dribbled from the meatus (Fig. 1). Blood biochemistry was normal. Ultrasound and voiding cystourethrogram showed a large diverticulum located in anterior urethra (Fig. 2). There were no vesicoureteral reflux and back-pressure changes. Open diverticulectomy and urethroplasty was done. A midline incision was made over the diverticulum, which was dissected up to the neck till the normal corpus spongiosum was visible. The diverticulum was then opened in the midline. This incision was continued distally across the distal lip of the diverticulum to divide the attenuated tissue in this region. A triangular flap to fit the inverted `V'-shaped defect of the incised distal lip was fashioned from one-half of the diverticulum and the remaining wall was excised. From the other half of the diverticulum a rectangular flap was fashioned in order to double-breast the urethral suture line after scraping of the mucosa with the flat of a knife. The urethra was catheterized with a 6fr infant feeding tube, which was retained as a urethral stent for 10 days. Postoperatively the boy passed urine in a good stream without straining and discomfort. There was no swelling at the site of the diverticulum. Urethral calibration was done by the smooth passage of a 10fr infant feeding tube 4 weeks after the procedure. The patient was followed up at 3-monthly intervals for 1 year, and he remained symptom free.

**Figure:**
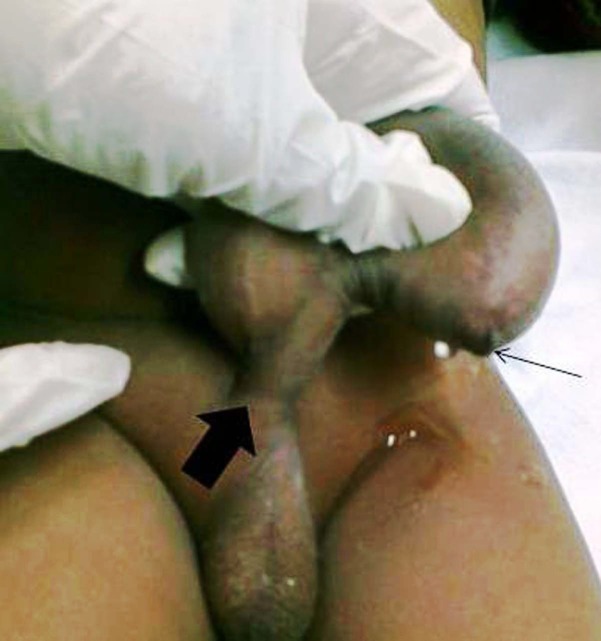
Figure 1:Compressible penile swelling with urine dribbling from the meatus. Thick arrow: penoscrotal junction, Thin arrow: meatus with dribbling of urine

**Figure:**
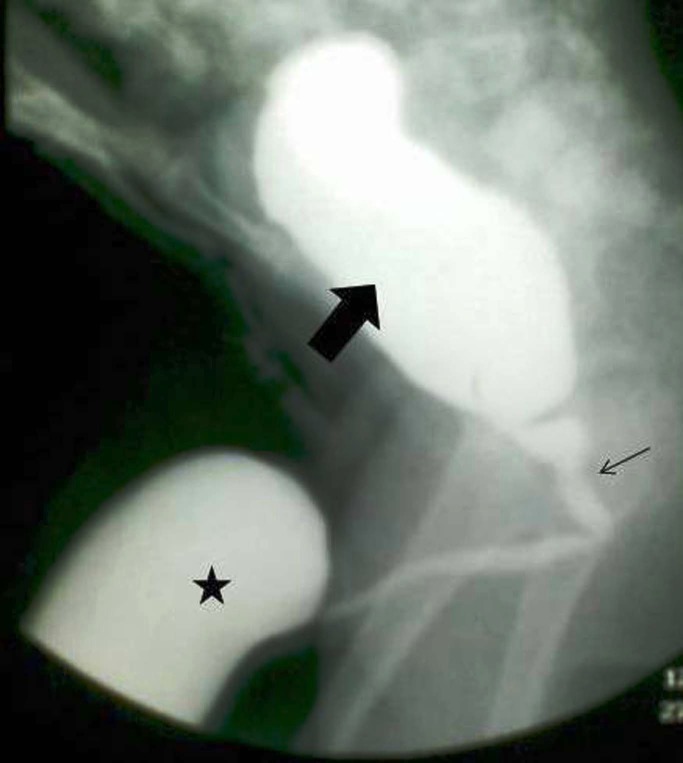
Figure 2:Voiding cystourethrogram showing the anterior urethral diverticulum. Thick arrow: bladder, Thin arrow: posterior urethra, Asterix: diverticulum in anterior urethra

CAUD accounts for only 10%-20% of all diverticulae of the anterior urethra and composed of three types: wide-mouthed diverticulae, narrow-mouthed diverticulae, and megalourethra, which is a generalized dilatation of the entire anterior urethra.[2] The wide-mouthed diverticulae are usually saccular and narrow mouthed diverticulae are mostly spherical. A wide-mouthed diverticulum may or may not have a distal lip. As urine fills the diverticulum during voiding, the distal lip elevates and presses against the urethra causing obstruction. In the present case, the diverticulum was spherical in shape with narrow distal lip.

CAUD patients usually present with penile swelling, recurrent urinary tract infection and poor urinary stream as in the present case. CAUD can be diagnosed antenatally by ultrasound. Marsupialization is an excellent option for temporary urinary diversion when the clinical situation precludes primary excision and repair. Subsequent diverticulectomy and urethroplasty can be done at 6 months of age.[3]

CAUD patients usually present with penile swelling, recurrent urinary tract infection and poor urinary stream as in the present case. CAUD can be diagnosed antenatally by ultrasound. Marsupialization is an excellent option for temporary urinary diversion when the clinical situation precludes primary excision and repair. Subsequent diverticulectomy and urethroplasty can be done at 6 months of age.[3]

## Footnotes

**Source of Support:** Nil

**Conflict of Interest:** None declared

